# Association between transported Asian dust and outdoor fungal concentration during winter in a rural area of western Japan

**DOI:** 10.1186/s41021-017-0079-7

**Published:** 2017-07-01

**Authors:** Kyoko Iwata, Masanari Watanabe, Jun Kurai, Naoto Burioka, Sachiko Nakamoto, Degejirihu Hantan, Eiji Shimizu

**Affiliations:** 1 Department of Respiratory Medicine and Rheumatology, Faculty of Medicine, Tottori University, Tottori, Japan; 2 Mio Fertility Clinic, Reproductive Centre, Tottori, Japan; 3 Division of School of Health Science, Department of Pathobiological Science and Technology, Tottori University Faculty of Medicine, Tottori, Japan

**Keywords:** Airborne particulate matter, Asian dust, Cytokine production, Light detection and ranging, Outdoor fungi

## Abstract

**Background:**

Recently, Asian dust (AD) has become a serious health problem and several studies have clearly proven that AD can aggravate asthma. However, it remains unclear as to which components of AD have a strong effect on the asthma exacerbation caused by AD exposure. Outdoor fungi can increase emergency department visits and hospitalization for asthma exacerbation and can aggravate asthma symptoms. Therefore, this study was aimed at investigating the relationship between AD and outdoor fungi and determining the potential of fungi to cause airborne particulate matter (PM)-related inflammatory responses.

**Methods:**

Airborne PM was collected each day from January 26, 2015 to February 27, 2015. Daily levels of outdoor fungi-associated PM were calculated using a culture-based method. Production of cytokines such as interleukin (IL)-6, IL-8, and tumor necrosis factor (TNF)-α was assessed in THP1 cells stimulated by the collected airborne PM each day.

**Results:**

Daily levels of AD particles were assessed using Light Detection and Ranging and did not correlate with outdoor fungi (r = −0.17, *P =* 0.94). There was also no association between outdoor fungi and the daily production of IL-6 (r = 0.16, *P =* 0.37), IL-8 (r = 0.19, *P =* 0.30), or TNF-α induced by collected PM (r = 0.07, *P =* 0.70). However, the daily levels of AD particles were significantly associated with IL-6 (r = 0.91, P < 0.0001), IL-8 (r = 0.64, *P =* 0.0004), and TNF-α (r = 0.72, *P* < 0.0001) production.

**Conclusion:**

AD did not increase the acute levels of outdoor fungi and outdoor fungi did not affect the cytokine production induced by airborne PM. These results suggest that outdoor fungi do not have any detectable effect on the asthma exacerbation caused by AD exposure.

## Background

A large emission of sand dust in East Asia is referred to as Asian dust (AD), which originates mostly in the deserts of Central Asia, China, and Mongolia. AD is driven across the Korean Peninsula to Japan by prevailing westerly winds and provokes a yellow fog often in late winter and early spring [1]. A growing number of studies in epidemiology, toxicology, and other related fields have indicated that AD is closely related to the incidence of human diseases and mortality rate [2–7]. AD is also reported to increase the risk of exacerbation of asthma [8–13].

AD is composed of not only natural materials (soil components) but also anthropogenic particles such as sulfate oxide and nitrate oxide derived from fossil-fuel power stations and motor vehicles [14, 15]. Although AD has been suggested to exert allergic and toxic effects, it remains uncertain as to which components of AD are associated with the exacerbation of asthma. Several studies have reported that outdoor fungi can increase emergency department visits and hospitalization for asthma exacerbation and can aggravate asthma symptoms [16–18]. AD has also been shown to carry microbial agents [19] and a few studies have reported increases in the abundance of several fungal taxa during heavy AD days [20–22]. Therefore, the fungi included in AD may constitute one of the causes for the exacerbation of asthma caused by AD.

Airway epithelial cells and inflammatory cells are the first to be exposed to airborne particulate matter (PM) such as AD particles. These cells are known to release a multitude of biochemical compounds including pro-inflammatory cytokines such as interleukin (IL)-6, IL-8, and tumor necrosis factor (TNF)-α in response to PM exposure [23]. Various components of PM can influence the subsequent pro-inflammatory cytokine response [24], with the inflammatory potential of PM being heterogeneous depending on the city and season [25–28]. Several studies have shown that these heterogeneous inflammatory responses may be partly attributed to the elemental composition of AD [25, 26, 29]. However, the effects of fungi in airborne PM on the inflammatory response remain unclear.

Our previous study showed that exposure to outdoor fungi was associated with pulmonary dysfunction in children [30]. However, the relationship between fungi and AD has not been extensively studied in Japan, and the components having strong effects on the asthma exacerbation caused by AD exposure have not been identified. Therefore, this study was aimed at investigating the relationship between the quantity of outdoor fungi and AD, because fungi associated with airborne PM may have a marked effect on asthma exacerbation caused by AD exposure. In the present study, we also evaluated the effects of fungi in airborne PM on inflammatory responses induced by airborne PM.

## Methods

### Air pollutant levels

The level of suspended particulate matter (SPM) is monitored at multiple locations throughout Japan by the Japanese Ministry of the Environment. Data for SPM collected in Matsue City were used in the present analysis. Light Detection and Ranging (LIDAR) systems can measure the levels of sand dust particles moving from East Asia to Japan, which can be used to identify non-spherical dust particles and spherical particles using laser light of two wavelengths within a height of <1 km above the ground level [31–33]. LIDAR systems measure the levels of non-spherical dust particles such as AD particles at 15-min intervals, and the daily levels are determined based on the median value of 96 measurements collected over a 24-h period from the midnight of one day to the midnight of the next day. LIDAR data from Matsue City from 120 to 150 m above the ground, which is the minimum altitude required by LIDAR systems, was obtained from the Japanese Ministry of the Environment. However, the definition of a heavy AD day using LIDAR measurements has not been established. According to a previous study by Ueda et al., a moderate Asian dust storm (ADS) day can be defined as a daily (24-h) average of 0.066 AD particles (non-spherical particles) per kilometer, and a heavy ADS day would show a daily average of 0.105 AD particles per kilometer [33]. Therefore, when the level of AD particles exceeds 0.06 km^−1^, it would be defined as a heavy ADS day.

### Calculation of the daily level of fungi-associated PM

From January 26, 2015, to February 27, 2015, the daily level of outdoor fungi-associated airborne PM was assessed in Matsue city, the capital city of the Shimane Prefecture in southwest Japan. Matsue city has a population of approximately 200,000 inhabitants and covers a geographical area of 530.2 km^2^. Total suspended particles were collected on a 20 × 25 cm quartz filter (2500QAT-UP; Tokyo Dylec, Tokyo, Japan) at a flow rate of 1000 L/min using a high-volume air sampler (HV-1000R; Shibata, Tokyo, Japan) for 23 h from 7 AM to 6 AM the following day. Before sampling, in order to relieve endotoxins from filters, the filters were sterilized by dry heat at 240 °C for 30 min. After sampling, the 4-cm^2^ filter was detached and extracted with 4 mL distilled deionized water. The daily level of outdoor fungi-associated airborne PM was calculated using the culture-based method [34, 35]. To culture fungi among the collected total suspended particles, 500 μL out of the 4 mL of filter extract was spread on Sabouraud agar in a 90-mm diameter dish. After 5 days of culture at 28 °C, the growing colonies were counted and the mean value of five dishes was calculated. The daily levels of outdoor fungi-associated PM were expressed as colony forming units per cubic meter of air (CFU/m^3^).

### Cell culture and measurement of IL-6, IL-8, and TNF-α release

After resection, the 4-cm^2^ filters used for fungal culture were extracted with 4 mL endotoxin-free distilled deionized water (sterile endotoxin-free water; Wako Pure Chemicals, Osaka, Japan) using an ultrasonic apparatus (BRANSONIC2800; Emerson Japan, Atsugi, Japan) for 60 min. These extraction liquids were filtered through 10-μm filters (pluriStrainer 10 μm; pluriSelect, Leipzig, Germany) to remove PM with sizes > 10 μm. PM < 10 μm in diameter dissolved in the solution, which was then sterilized at 121 °C for 30 min in an autoclave (Tomy SX-300; Tomy, Tokyo, Japan) and stored in a freezer at −70 °C to prevent the growth of bacteria and fungi.

THP1 (ATCC^®^ TIB-202™) human monocyte cell lines were cultured in Roswell Park Memorial Institute medium 1640 containing 10% (v/v) fetal bovine serum, 0.05 mM 2-mercaptoethanol, 100 U/mL penicillin, 100 μg/mL streptomycin, and 0.5 μg/mL amphotericin B at 37 °C and 5% CO_2_ in a humidified cell culture incubator. For the negative control, the THP1 cells (1 × 10^5^ cells/450 μL/tube) were exposed to solvent only for 24 h at 37 °C in an endotoxin-free tube (pirotube; Seikagaku, Tokyo, Japan), and the test cells were added to 50 μL of each PM solution. After exposure, the culture supernatants were removed and first centrifuged at 250 × *g* to remove the floating cells and then at 2,500 × *g* to remove the remaining particles. The final supernatants were stored at −70 °C. The concentrations of IL-6, IL-8, and TNF-α in the cells were then measured using an enzyme-linked immunosorbent assay (ELISA) kit for IL-6, IL-8, and TNF-α (R&D Systems, Minneapolis, MN, USA) according to the manufacturer’s protocols with endotoxin-free 96-well plates (Toxipet plateLP; Seikagaku, Tokyo, Japan). The samples were run in triplicate and read using an automated ELISA reader (Model 680, Bio-Rad, Philadelphia, PA, USA). The production levels of IL-6, IL-8, and TNF-α induced by each dissolving solution were defined as the daily IL-6, IL-8, and TNF-α levels induced by SPM.

### Statistical analysis

The associations between fungal levels and the daily levels of SPM, AD particles, IL-6, IL-8, and TNF-α were assessed by linear regression analysis. Data analyses were performed using SPSS statistical software (Japanese ver. 21.0 for Windows; IBM Japan, Tokyo, Japan). All P-values were two-sided with a significance level of 0.05.

## Results

### Levels of SPM, AD particles, and outdoor fungi

**Fig. 1:**
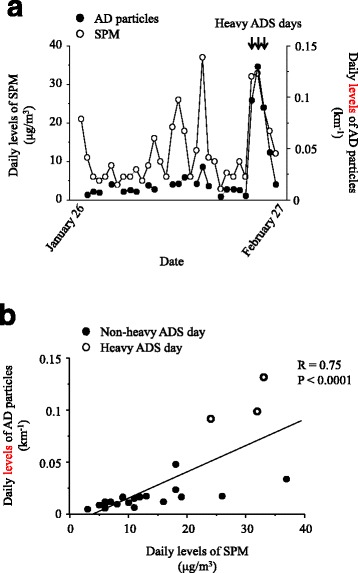
(**a**) Daily levels of suspended particulate matter (SPM) (*open circles*) and Asian dust (AD) particles (*closed circles*) from January 26, 2015 to February 27, 2015. The daily levels of AD particles from East Asia to Japan were measured using Light Detection and Ranging (LIDAR) systems. A heavy ADS day was defined as a day when the level of AD particles exceeded 0.06 km^−1^. (**b**) Associations between daily levels of SPM and AD particles. Closed circles represent non-heavy ADS days and open circles indicate heavy ADS days

Figure 1a shows the daily levels of SPM and AD particles from January 26 to February 27, 2015. The data of AD particles were lacked in eight days: January 26, January 30, and February 1, 5, 8, 9, 13, and 17. The mean levels of SPM and AD particles were 12.7 ± 9.0 μg/m^3^ and 0.02 ± 0.03 km^−1^, respectively. The daily levels of the AD particles exceeded the 0.6 km^−1^ threshold on February 23, 24, and 25 (0.10, 0.13, and 0.9 km^−1^, respectively); these three days were therefore determined as heavy ADS days. Figure 1b shows the significant correlation between the daily levels of SPM and AD particles.

**Fig. 2:**
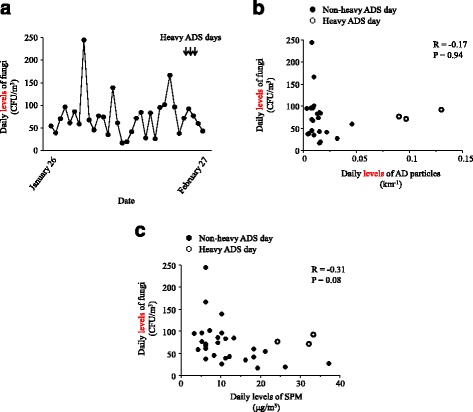
(**a**) Daily levels of fungi associated with airborne particulate matter from January 26 to February 27, 2015. (**b**) Associations between daily levels of fungi and Asian dust (AD) particles. (**c**) Associations between daily levels of fungi and suspended particulate matter (SPM). Closed circles represent non-heavy ADS days and open circles indicate heavy ADS days (**b**, **c**)

Figure 2a presents the daily levels of outdoor fungi; the average level was 73.5 ± 44.7 CFU/m^3^ from January 26 to February 27, 2015. The levels of fungi on the heavy ADS days were as follows: 72.2 CFU/m^3^ on February 23, 93.1 CFU/m^3^ on February 24, and 77.1 CFU/m^3^ on February 25. The daily levels of AD particles exceeded the mean level on two of these three heavy ADS days. There was no correlation between the daily levels of SPM, AD particles, and outdoor fungi (Fig. 2b and c).

### Associations between pro-inflammatory cytokine production, outdoor fungi, and AD particles

**Fig. 3:**
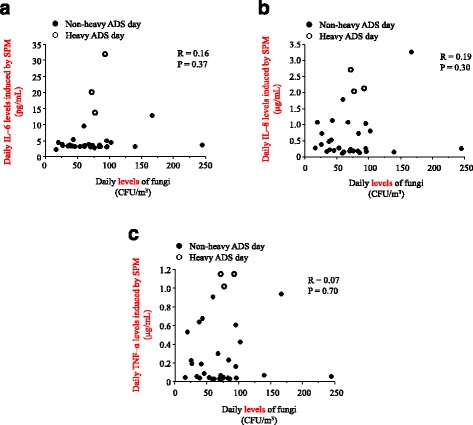
Associations between daily levels of fungi and (**a**) interleukin (IL)-6, (**b**) IL-8, and (**c**) tumor necrosis factor (TNF)-α levels induced by suspended particulate matter (SPM). Closed circles represent non-heavy ADS days and open circle indicate heavy ADS days

**Fig. 4:**
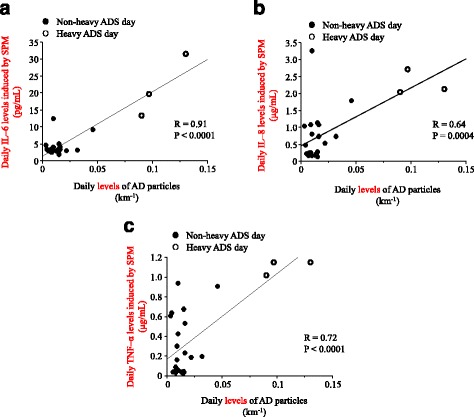
Associations between daily levels of Asian dust (AD) particles and (**a**) interleukin (IL)-6, (**b**) IL-8, and (**c**) tumor necrosis factor (TNF)-α levels induced by suspended particulate matter (SPM). Closed circles represent non-heavy ADS days and open circles indicate heavy ADS days

The pH levels of all the collected airborne PM-dissolving solutions ranged from 7.6 to 8.1. After stimulation by the collected airborne PM, the viability of the THP1 cells exceeded 95% in all the samples, as assessed using a trypan blue-exclusion test. However, we did not find any association between the daily IL-6, IL-8, and TNF-α levels induced by SPM and the daily levels of outdoor fungi (Fig. 3a, b, and c). In contrast, there were significant relationships between the daily IL-6, IL-8, and TNF-α levels induced by SPM, and the daily levels of AD particles (Fig. 4).

## Discussion

Although little is known regarding the association between outdoor fungi and AD in Japan, studies conducted in South Korea and Taiwan have shown that AD can contribute to the amount and characteristics of outdoor fungi [20–22, 36]. In these studies, the researchers used the levels of SPM or particulate matter smaller than 10 μm in aerodynamic diameter (PM_10_) to determine a heavy ADS day. Similarly, most of the research on AD often involved the use of SPM or PM_10_ to determine a heavy ADS day. Recently, it has become possible to measure the daily quantity of AD particles using LIDAR systems. According to LIDAR data, small quantities of AD particles arrive in Japan mostly from February to May [31, 32]. To the best of our knowledge, the present study is among the first to use LIDAR data to study the association between the quantity of outdoor fungi and AD.

In the present study, we did not find any association between the daily levels of outdoor fungi and AD particles. Similarly, there was no relationship between the daily levels of outdoor fungi and SPM. We were unable to analyze the differences in the amount of outdoor fungi between heavy ADS days and non-heavy ADS days, because there were only three heavy ADS days during the study period. This also compromised our ability to identify the association between the levels of outdoor fungi and AD particles. The effects of AD on the amount of outdoor fungi vary between studies, even when the studies are conducted in the same country. For example, in Taiwan, Ho et al. found that heavy levels of AD could increase the concentrations of outdoor fungi [20]. They also showed a significant positive association between the levels of outdoor fungi and SPM. However, Wu et al. were unable to find the same effect in Taiwan [21]. Furthermore, in another study, Alghamdi et al. found an association with inverse PM2.5 concentrations (1/PM2.5) and outdoor fungi [37], which may indicate that the fungal levels do not correlate with PM mass levels. Alghamdi et al. also suggested that wind speed positively correlates with the levels of bacteria and fungi-associated airborne PM [37]. Therefore, in order to positively define the effects of AD on the levels of outdoor fungi, further studies need to be conducted taking wind into consideration.

Considerable evidence indicating an association between the exacerbation of asthma and AD has been accumulated [8–13]. However, the mechanism underlying the AD-induced exacerbation of asthma remains unclear. Several studies have shown that outdoor fungi were associated with an increase of emergency department visits, hospitalizations, and aggravation of asthma symptoms in patients with asthma [16–18]. However, according to the results of the present study, heavy exposure to outdoor fungi does not represent a likely cause for the exacerbation of asthma caused by exposure to AD.

A multitude of in vitro studies have focused on the effects of airborne PM on pro-inflammatory cytokine production to understand the mechanisms underlying the adverse health effects caused by exposure to airborne PM [23]. However, researchers have found it difficult to identify the components of airborne PM that have the most effect on cytokine production. Our previous study showed that the effects of airborne PM exposure on pulmonary function in schoolchildren differ with the type and source of the PM [38]. In contrast, in the present study, we were unable to find any association between outdoor fungi and the production of cytokines such as IL-6, IL-8, and TNF-α in THP1 cells exposed to airborne PM. However, we found significant relationships between the production of cytokines such as IL-6, IL-8, and TNF-α, and the level of AD particles. Based on this result, we believe that exposure to AD particles might exacerbate asthma through the aggravation of airway inflammation. However, outdoor fungi were not found to play a role in the association between inflammatory response and AD exposure.

In order to investigate the underlying mechanism by which airborne PM provokes an adverse effect on health, a multitude of in vitro studies have been performed focusing on cytokine production. In a review by Mitschik et al. on the in vitro effects of airborne PM on cytokine production [23], TNF-α was revealed as the cytokine most often selected for revealing details about the harmful effects of airborne PM on health, followed by IL-8 and IL-6. This result underscores the proposed key roles of TNF-α, IL-8, and IL-6 in the inflammatory responses to airborne PM. Therefore, in the present study, TNF-α, IL-8, and IL-6 were selected to examine the relationship between fungi in airborne PM on the inflammatory responses induced by airborne PM. However, in 27 out of 33 days during study period, the levels of IL-6 were below 5 pg/mL. Compared to TNF-α and IL-8, the sensitivity was thus insufficient to study the effect of PM on IL-6 production owing to the small amount of collected PM in this study.

To evaluate the inflammatory responses induced by collected SPM in the present study, we were desirous of using dendritic cells (DCs), which populate the airways and play important roles in antigen presentation, cytokine production, and the initiation of inflammation. In particular, inhalation substances contact DCs in the airway subsequent to initial contact with airway epithelial cells. However, the culture procedures necessary to generate DCs from human monocytes are costly and time consuming. Alternatively, human monocyte cell lines have been explored as potential surrogates for human DCs [39], with THP1 cells being reported to exhibit potential as DC surrogates [40]. Therefore, the present study investigated the inflammatory responses induced by collected SPM using the THP1 cell line.

There are several limitations of this study. First, outdoor gaseous and particulate data generally and largely depend on season, weather, and region. The present study was not conducted across multiple locations, seasons, and years. Therefore, the results of the present study were unable to confirm the reproducibility of the association between AD and outdoor fungal concentration. Second, the culture-based method used for calculating outdoor fungi was unable to detect all existing outdoor fungi, because several strains of fungi cannot grow on Sabouraud agar culture. Therefore, it is reasonable to conclude that we underestimated the concentrations of fungi. Third, the present study did not include investigation of the characteristics of the fungi. Fourth, the daily amount of airborne PM was very small compared to the filter weight. Therefore, measurement of the quantity of daily collected airborne PM lacked precision. This resulted in an inability to calculate the daily levels of IL-6, IL-8, and TNF-α to obtain a unit quantity of collected airborne PM.

## Conclusion

In this study, we used a LIDAR system to determine the daily levels of SPM, AD particles, and outdoor fungi. No association was identified between the daily levels of outdoor fungi and AD particles. We also did not find any evidence of an increase in the levels of outdoor fungi caused by AD arriving from East Asia to Japan. Additionally, outdoor fungi did not appear to affect the cytokine production induced by exposure to airborne PM. However, we did find significant relationships between the production of IL-6, IL-8, and TNF-α and the level of AD particles. These results suggest that outdoor fungi are unlikely to exert any noticeable effect on the asthma exacerbation caused by AD exposure.

## Abbreviations

AD: Asian dust
ADS: Asian dust storm
CFU/m^3^: Colony forming units per cubic meter of air
ELISA: Enzyme-linked immunosorbent assay
IL: Interleukin
LIDAR: Light Detection and Ranging
PM: Particulate matter
SPM: Suspended particulate matter
TNF-α: Tumor necrosis factor-α
